# An *Arabidopsis* soluble chloroplast proteomic analysis reveals the participation of the Executer pathway in response to increased light conditions

**DOI:** 10.1093/jxb/erv018

**Published:** 2015-03-04

**Authors:** Estefanía Uberegui, Michael Hall, Óscar Lorenzo, Wolfgang P. Schröder, Mónica Balsera

**Affiliations:** ^1^Instituto de Recursos Naturales y Agrobiología, Consejo Superior de Investigaciones Científicas (IRNASA-CSIC), 37008-Salamanca, Spain; ^2^Department of Chemistry, Umeå University, SE-90187 Umeå, Sweden; ^3^Centro Hispano Luso de Investigaciones Agrarias (CIALE), Universidad de Salamanca, 37185 Salamanca, Spain

**Keywords:** Abiotic stress, acclimation response, chloroplast metabolism, DIGE, light, retrograde signalling, ROS.

## Abstract

The Executer1 and Executer2 proteins have a fundamental role in the signalling pathway mediated by singlet oxygen in chloroplast; nonetheless, not much is known yet about their specific activity and features. Herein, we have followed a differential-expression proteomics approach to analyse the impact of Executer on the soluble chloroplast protein abundance in *Arabidopsis*. Because singlet oxygen plays a significant role in signalling the oxidative response of plants to light, our analysis also included the soluble chloroplast proteome of plants exposed to a moderate light intensity in the time frame of hours. A number of light- and genotype-responsive proteins were detected, and mass-spectrometry identification showed changes in abundance of several photosynthesis- and carbon metabolism-related proteins as well as proteins involved in plastid mRNA processing. Our results support the participation of the Executer proteins in signalling and control of chloroplast metabolism, and in the regulation of plant response to environmental changes.

## Highlight

Executer proteins have emerged as participants in singlet oxygen-mediated retrograde signalling in plants. This study relates the function of Executer in chloroplasts with the acclimation response to increased light.

## Introduction

During evolution, plants have developed an intricate network of signalling pathways to trigger physiological responses as a consequence of diverse environmental stimuli. Recent observations have demonstrated that retrograde communication coordinates the expression of nuclear genes with the metabolic and developmental state of the cell through signals emitted from plastids and mitochondria (Woodson and Chory, 2008; Ng *et al.*, 2014). Particularly, chloroplasts─photosynthetic organelles in plant cells─are the source of specific signalling molecules that relay information to the nucleus (Nott *et al.*, 2006; Pfannschmidt, 2010; Pogson and Albrecht, 2011; Barajas-López *et al.*, 2013; Chi *et al.*, 2013). The plastid signals identified so far can be linked to specific stress conditions, and the best characterized signals are intermediates of the tetrapyrrole biosynthesis pathway, the redox state of the thylakoid membrane, and reactive oxygen species (ROS) (Karpinski *et al.*, 1999; Strand *et al.*, 2003; Gadjev *et al.*, 2006; Koussevitzky *et al.*, 2007; Foyer and Noctor, 2009; Galvez-Valdivieso *et al.*, 2009; Pesaresi *et al.*, 2009; Sun *et al.*, 2011; Woodson *et al*., 2011; Zhang *et al.*, 2011; Pfalz *et al.*, 2012; Petrillo *et al.*, 2014). Also, the metabolic state of chloroplasts can be sensed by exported metabolites such as carbohydrates, reductive power in the form of NADPH, isoprenoid intermediates, xantophyll derivatives or a phosphonucleotide (Rolland *et al.*, 2006; Pfannschmidt, 2010; Zhang *et al.*, 2010; Estavillo *et al.*, 2011; Ramel *et al.*, 2012; Xiao *et al.*, 2012).

In chloroplasts, ROS are produced as an unavoidable side effect of the photosynthetic light reactions, as a consequence of the spatial and temporal concurrence of electron and energy transfer reactions with photosynthetically generated molecular oxygen (Asada, 2006; Galvez-Valdivieso and Mullineaux, 2010). Whereas electron transfer reactions to molecular oxygen in its ground triplet state lead to the formation of superoxide, hydrogen peroxide and hydroxyl radicals, energy transfer reactions result in the formation of the highly reactive singlet oxygen. ROS generation in chloroplasts is enhanced during environmental stress, and particularly singlet oxygen is the major ROS produced in leaves exposed to high light (Fryer *et al.*, 2002; Krieger-Liszkay *et al.*, 2008). Genetic approaches have shown that the production of singlet oxygen under controlled conditions in the chloroplast plays a significant role in signalling the oxidative response of plants (Apel, 2004). Subsequent work in *C. reinhardtii* showed that earlier exposure of the cells to low levels of singlet oxygen elicits an acclimation response that protects cells from photooxidative damage (Ledford *et al.*, 2007).

However, the mechanism which transduces the signal from the chloroplast to the nucleus is far from delineated and, in higher plants, appears to undergo significant crosstalk with other signalling pathways controlling such responses as plastid differentiation, plant development and general stress responses (Laloi *et al.*, 2007; Baruah *et al.*, 2009). With the aim of studying the biological activity of singlet oxygen and the mechanisms involved in transduction of redox signalling, the conditional *flu* mutant of *Arabidopsis thaliana* was identified (Meskauskiene *et al.*, 2001). The *flu* mutant accumulates free protochlorophyllide (Pchlide), the immediate precursor of chlorophyllide, in the dark; upon illumination free Pchlide acts as a potent photosensitizer that generates singlet oxygen by transferring light energy to molecular oxygen (Gollnick, 2007). Two stress responses in *flu* were triggered by the release of singlet oxygen within the plastid compartment during re-illumination of dark-adapted plants: seedling lethality, and cell death and growth inhibition in mature plants (op den Camp *et al.*, 2003). Global gene-expression studies showed that these stress responses were not primarily due to physicochemical damage caused by singlet oxygen during oxidative stress but were attributed to the activation of genetic stress response programs (op den Camp *et al.*, 2003; Gadjev *et al.*, 2006; Laloi *et al.*, 2006; Przybyla *et al.*, 2008). An extensive second-site mutant screen performed in *flu* identified a group of suppressor mutants named *executer* (*ex*). Plants with mutations in Executer1 (Ex1), a plastid protein of unknown function, lost the ability to perceive the presence of singlet oxygen in *flu* chloroplasts and, consequently, the activation of singlet-oxygen mediated response programs was suppressed (Wagner *et al.*, 2004). Subsequently, a protein homolog to Ex1, named Executer2 (Ex2), was also identified and suggested to be involved in the singlet-oxygen-responsive gene network (Lee *et al.*, 2007).

How the Executer proteins are involved and function in the signalling pathway remains elusive. The loss of function of either of the Executer protein showed no obvious phenotype compared to WT *Arabidopsis* (Lee *et al.*, 2007; Wagner *et al.*, 2004; Kim and Apel, 2013*a*). It was found however that *ex1* plants were more resistant than WT to damage upon treatment with low concentrations of 3-(3, 4-dichlorphenyl)-1,1-dimethylurea (DCMU) together with high light intensities (Wagner *et al.*, 2004). Also, analysis of the response of WT and *ex1* plants to β-cyclocitral (a β-carotene oxidation product) treatments suggested that *ex1* plants were more resistant to photooxidative stress than WT (Ramel *et al.*, 2012). Studies on the hypersensitive response to pathogen infection pointed out that *ex2* plants are slightly more resistant to low amounts of pathogens than WT (Mur *et al.*, 2010). Furthermore, a double mutant *ex1/ex2* was affected in chloroplast development in cotyledons (Kim *et al.*, 2009), and the seedlings of the double mutant *ex1/ex2* were less susceptible than WT when exposed to a combined low-temperature/high-light treatment (Kim *et al.*, 2012).

The role of Executer proteins in singlet-oxygen mediated signalling is unclear. Because light availability is one of the key factors that modulates acclimation strategies and defence reactions in plants, we aim to analyse how plants adapt to their environment by studying the chloroplast proteome response to a perturbation in light intensity, which promotes ROS production but would not result in oxidative stress or cell death. Here, a differential-expression proteomics approach was used to analyse the impact of light on chloroplast protein abundance in two T-DNA insertional knockout lines (*EX1* and *EX2*). Our study showed changes in abundance of several photosynthesis- and carbon metabolism-related proteins as well as proteins involved in plastid mRNA processing, among others. A good correlation between *executer* mutants and the changes occurring after exposure of WT plants at a moderate light intensity in the time frame of hours was inferred. It is suggested that Executer proteins participate in signalling in *Arabidopsis* under growth light conditions, and in the regulation of the response to environmental cues such as light acclimation, likely to avoid the misexpression of defence programs.

## Material and methods

### Plant materials

For all the experiments, *Arabidopsis thaliana* plants (WT and mutants) of the ecotype Columbia (Col-0) were used. The SALK_002088C and SALK_021694C lines─harbouring a T-DNA insertion in the *EX1* (At4g33630) and *EX2* (At1g27510) genes, respectively─were purchased from NASC (Nottingham Arabidopsis Stock Center) (Alonso *et al.*, 2003). Plants were grown on soil or on MS basal salt medium. Genomic DNA was isolated from leaf material using the CTAB extraction protocol adapted from Weigel (2002) and screened for T-DNA insertion by PCR genotyping. The following genomic primers were used: *EX1* forward gene specific primer (FP; 5′-CACTCCCTCCTCCAAAAGATC-3′) and *EX1* reverse gene specific primer (RP; 5′-TACCCCAATCACTCAAATTGG-3′) to characterize insertion lines SALK_002088; *EX2*-FP (5′-CACTAAGCT TGTCATCGGAGG-3′) and *EX2*-RP (5′-AAATGTCAATGTG GCTGGAAC**-**3′), to characterize insertion lines SALK_021694. In these experiments, the T-DNA–specific left border (LB) primer LB (5′-ATTTTGCCGATTTCGGAAC-3′) was also used. To verify PCR products and T-DNA insertion sites, amplified DNA fragments were sequenced.

### Light treatment

Plants were grown on soil in a growth chamber at 8 h-light/16 h-darkness (20ºC) for 7–8 weeks under a photon flux density (PFD) of 120μmol m^-2^ s^-1^ and relative humidity of 70%. For the high light (HL) experiments, plants were transferred 1h after the onset of the light period to a growth chamber under PFD of 600–700μmol m^-2^ s^-1^. As control material one set of plants was maintained at 120μmol quanta m^-2^ s^-1^, representing normal light (NL) conditions.

### Protein extraction

After 6h light treatment (HL or NL), plant leaves were harvested for chloroplast isolation and purification according to Hall *et al.* (2011). Briefly, 20g of plant material was homogenized using a blender in ice-cold extraction buffer (20mM Tricine-NaOH pH 8.4, 300mM sorbitol, 10mM KCl, 10mM Na-EDTA, 0.25% BSA, 4.5mM sodium ascorbate and 5mM L-cysteine). Cell debris was removed by a nylon mesh (22μm), and chloroplasts were pelleted by centrifugation for 2min at 1000 ×*g*. Chloroplasts were washed and ruptured by osmotic shock in 10mM Na-pyrophosphate-NaOH pH 7.8 buffer. Following centrifugation at 100 000×*g* for 1h at 4ºC, the supernatant containing the soluble stromal proteins was concentrated using an Amicon Ultra-15 10 K ultrafiltration device. Protein concentration was determined using the Bradford assay (Bradford, 1976) and bovine serum albumin as reference. Seppro® Rubisco Spin Columns (Sigma) were used to reduce Rubisco abundance in the stroma samples.

### Two-dimensional differential gel electrophoresis (2D-DIGE)

Chloroplast stroma samples were precipitated with ice cold acetone. Protein pellets were solubilized in DIGE labelling buffer (30mM Tris-HCl pH 8.5, 2M thiourea, 7M urea, 2% (w/v) CHAPS). Remaining insoluble material was removed by centrifugation for 10min at 21 000×*g*. The final protein concentration for G-Dye labelling was 5μg/μl. Solubilized protein samples were separately labelled with G-Dye100, G-Dye200 and G-Dye300 dyes (DyeAGNOSTICS) at a ratio of 400pmol dye/50µg protein extract for 30min in darkness on ice. Labelling was quenched by addition of lysine. In general, samples were labelled using G-Dye200 and G-Dye300 dyes while an internal standard (consisting of a pooled sample comprising an equal amount of all samples in the experiment) was labelled with G-Dye100. Details of labelling and the subsequent combination of differentially labelled samples used are shown in the experimental design presented in Table 1. For each immobilized pH gradient (IPG) strip, equal amounts of G-Dye100, G-Dye200 and G-Dye300 labelled samples were combined, typically 50µg protein per sample. Prior to iso-electric focusing the mixed samples were diluted with rehydration solution containing 2M thiourea, 7M urea, 2% (w/v) CHAPS, 20mM DTT, 0.002% (w/v) bromophenol blue and 0.5% (v/v) IPG buffer pH 3–11NL (GE Healthcare, Uppsala, Sweden). Samples were thereafter applied to 24cm Immobiline Dry Strips pH 3–11 NL (GE Healthcare, Uppsala, Sweden) by passive rehydration for 2h followed by active rehydration for 10h at 30V. Isoelectric focusing (IEF) was performed on an IPGphor II (GE Healthcare, Uppsala, Sweden). Prior to second dimension SDS-PAGE, strips were equilibrated first for 15min in 75mM Tris-HCl pH 8.8, 6M urea, 2% (w/v) SDS, 30% (v/v) glycerol, 0.002% (w/v) bromophenol blue and 1% (w/v) DTT and secondly for 15min in 75mM Tris-HCl pH 8.8, 6M urea, 2% (w/v) SDS, 30% (v/v) glycerol, 0.002% (w/v) bromophenol blue and 2.5% (w/v) iodoacetamide. Strips were applied on top of 12% SDS-polyacrylamide gels and sealed in place using 0.5% (w/v) agarose. Second dimension separation was performed using an Ettan Daltsix electrophoresis unit (GE Healthcare). Preparative samples for spot picking and subsequent mass spectrometry analysis, corresponding to 500 µg of protein per IPG strip (comprising an equal mixture of all samples in the experiment), were precipitated in acetone. Proteins were solubilised in 2M thiourea, 7M urea, 2% CHAPS, 20mM DTT, 0.002% (w/v) bromophenol blue and 0.5% (v/v) IPG buffer pH 3–11 NL (GE Healthcare, Uppsala, Sweden) before being separated by 2D-gel electrophoresis as described for DIGE gels above. Preparative gels were fixed in 30% ethanol, 10% acetic acid and were stained by Coomasie Brilliant Blue (CBB).

**Table 1.** Dye swap setup of the DIGE experiments using G-Dye100, G-Dye200 and G-Dye300. The experiment consisted of three genotypes (*ex1* and *ex2* mutant plants, and WT) and two light treatments (NL and HL) making a total of six groups. Four replicates were taken per sample for a total of 24 protein samples in the experiment. The internal standard contained equal amounts of protein extracts from all samples.

Gel	G-Dye100	G-Dye200	G-Dye300
**1**	Pooled standard	WTNL	*ex1*NL
**2**	Pooled standard	WTNL	*ex1*NL
**3**	Pooled standard	*ex1*NL	WTNL
**4**	Pooled standard	*ex1*NL	WTNL
**5**	Pooled standard	WTHL	*ex1*HL
**6**	Pooled standard	WTHL	*ex1*HL
**7**	Pooled standard	*ex1*HL	WTHL
**8**	Pooled standard	*ex1*HL	WTHL
**9**	Pooled standard	*ex2*NL	*ex2*HL
**10**	Pooled standard	*ex2*NL	*ex2*HL
**11**	Pooled standard	*ex2*HL	*ex2*NL
**12**	Pooled standard	*ex2*HL	*ex2*NL

### Gel image analysis

G-Dye labelled samples were visualized using a Typhoon™ 9400 Variable Mode Imager (GE Healthcare, Uppsala, Sweden). All gel images were scanned at 100µm resolution using a photomultiplier tube (PMT) voltage optimal for maximal pixel intensity without spot saturation. Prior to image analysis the gel images were cropped using ImageQuant™ v.5.2 (GE Healthcare, Uppsala, Sweden) in order to remove extraneous areas. DIGE analysis was performed using Redfin 3 software (Ludesi) as was matching of preparative CBB stained gels to DIGE gels. Images of CBB-stained gels were acquired using an image scanner and the Labscan software (GE Healthcare, Uppsala, Sweden). Spot detection, matching and statistical analysis was performed using the Redfin 3 program (www.ludesi.com). A principal component analysis (PCA) was performed to separate the gel samples according to their expression variation. One-way analysis of variance (ANOVA; p<0.001) and MannWhitney (p<0.05) tests were conducted to assess differential expression of protein abundance between the different groups. Minimum protein volume was set at 200 and differentially expressed proteins with a change in average spot volume of at least 2.0-fold were selected.

### In-gel digestion and protein identification

Spots of interest were excised from preparative gels using an Ettan Spotpicker™ spot picking station fitted with a 1.4mm picker head. Gel plugs were dehydrated and destained by incubation with a solution containing 20mM ammonium hydrogen carbonate in 35% acetonitrile. The solution was removed and gel pieces were dried by addition and removal of neat acetonitrile twice. Dried gel plugs were rehydrated on ice with 20mM ammonium hydrogen carbonate and 10% acetonitrile containing 2ng/µl trypsin (Promega). In-gel digestion was performed overnight at 37°C. Mass spectrometry analysis of in-gel digests was performed on a MALDI-TOF Voyager-DE™ STR Bio Spectrometry Workstation (Applied Biosystems). Database searches were performed on a local Mascot server licensed to Umeå University by Matrixscience (www.matrixscience.com), using *Arabidopsis* TAIR9 and Swiss-Prot databases. For searches a peptide mass error tolerance of 50ppm was accepted and carbamidomethylation of cysteine and oxidation of methionine were specified as variable modifications.

### Chlorophyll fluorescence measurements

*In vivo* chlorophyll *a* fluorescence was measured using a Dual-PAM-100 chlorophyll fluorescence photosynthesis analyser (Heinz Walz) on attached rosette leaves. After dark acclimation of the plants (15min), the measuring light (9µmol photons m^-2^ s^-1^) was turned on, and minimal fluorescence (*F*_*o*_) was determined. Leaves were exposed to a pulse of saturating light (3000µmol photons m^-2^ s^-1^, 0.8s) to determine the maximum fluorescence in the dark-adapted state (*F*_*m*_). Subsequently, leaves were illuminated with actinic red light at 660µmol of photons m^-2^ s^-1^ determining the steady-state level of fluorescence in the light (*F*_s_) and subjected to saturating pulses (0.8s) of 3000µmol photons m^-2^ s^-1^ for 20min to measure the maximal fluorescence in the light-adapted state (*F*_*m*_’). The maximum PSII efficiency was expressed as *F*_*v*_/*F*_*m*_ = (*F*_*m*_ − *F*_*o*_)/*F*_*m*_, and the PSII operating efficiency as Φ_PSII_ = (*F*_*m*_ *’ − F*_*s*_)/*F*_*m*_ *’* (Genty *et al.*, 1989). The NPQ coefficient was calculated using the Stern–Volmer equation, NPQ = (*F*_*m*_ − *F*_*m*_’)/*F*_*m*_’; 1 - *qP* was calculated as (*F*_*s*_ − *F*_*o*_’)/(*F*_*m*_^’^ − *F*_*o*_^’^) (Bilger and Björkman, 1990).

## Results

The aim of the present study was to examine the collective response of the soluble chloroplast proteome of 8-week-old *Arabidopsis* plants following a transition from normal light (NL) to moderate high light (HL, 5-fold increase in PFD) in the time scale of hours. Furthermore, using *Arabidopsis* mutant plants, the link between the Executer pathway and the acclimation response upon exposure of plants to high light was investigated.

### Photosynthetic performance in *ex1* and *ex2* single mutants is comparable to WT

The role of Executer in chloroplast light response was analysed using two independent T-DNA insertion lines inactivating *EXECUTER1* (*EX1*) and *EXECUTER2* (*EX2*), respectively. Absence of *Executer* transcripts was confirmed in the T-DNA insertion lines indicating them to represent true knockout lines (Supplementary Fig. S1). As previously reported (Wagner *et al.*, 2004; Lee *et al.*, 2007), *ex1* and *ex2* plants showed no obvious alterations of growth and development. Because Executer proteins have been associated with PSII (Kim and Apel, 2013*b*), PSII performance was assessed by measurement of chlorophyll-*a* fluorescence at room temperature in intact leaves from WT and *executer* mutant plants treated at the two different PFD (NL and HL) for 6h. No differences could be detected between WT and mutant plants, as deduced from the ratio of variable to maximum fluorescence (*F*_*v*_/*F*_*m*_), the quantum yield of PSII (Φ_PSII_), the degree of non-photochemical quenching (NPQ) and the excitation pressure of PSII (1-*qP*) (Table 2). HL treatment resulted in a 5% decline of *F*_*v*_/*F*_*m*_ for both WT and mutant plants.

**Table 2.** PSII performance of intact leaves from NL- or HL-treated WT and *executer* mutant plants was determined by chlorophyll-*a* fluorescence measurements using a PAM-fluorometer. The maximum quantum yield of PS II (*F*_*v*_/*F*_*m*_), the effective quantum yield of PSII (Φ_PSII_), the degree of non-photochemical quenching (NPQ) and the excitation pressure of PSII (1-*qP*) was deduced. Data represents mean *±* standard error. No significant differences were found between plants in the same light condition (ANOVA).

Parameter	WTNL	WTHL	*ex1*NL	*ex1*HL	*ex2*NL	*ex2*HL
***F***_***v***_ **/*F***_***m***_	0.82±0.01	0.78±0.02	0.83±0.01	0.78±0.01	0.83±0.01	0.75±0.04
**NPQ**	1.79±0.10	1.53±0.25	1.53±0.13	1.22±0.18	1.63±0.16	1.24±0.03
**Φ**_**PSII**_	0.18±0.02	0.16±0.02	0.15±0.03	0.19±0.02	0.18±0.02	0.16±0.04
**1-*qP***	0.72*±*0.04	0.73*±*0.04	0.76*±*0.05	0.68*±*0.02	0.72*±*0.02	0.72*±*0.05

### 2D-DIGE analysis of the soluble chloroplast proteome highlights molecular differences between *executer* mutants and WT plants

**Fig. 1.**
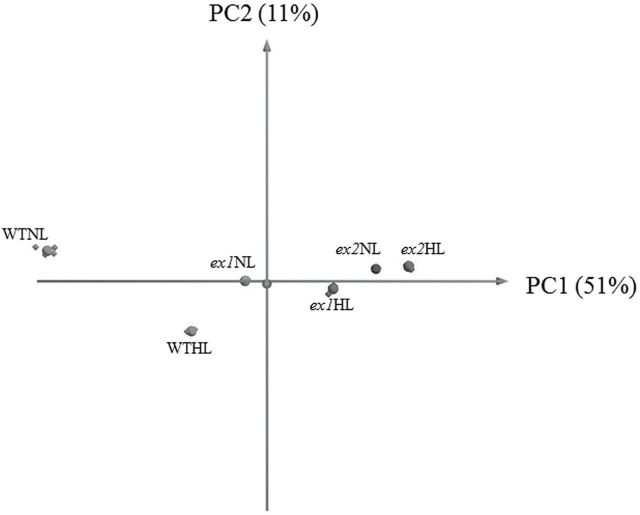
A principal component analysis (PCA) biplot for the complete data set demonstrated a distinct clustering of WT from the *executer* mutants (*ex1* and *ex2*) in the two light treatments (NL and HL). Principal components 1 (PC1) and 2 (PC2)—where PC1 indicates the direction of the highest variability followed by PC2 with diminishing variability orthogonal to PC1—accounted for 51% and 11% of the study variance, respectively. PCA suggested a modification of the soluble chloroplast proteome in *Arabidopsis* upon light treatment. The genotype caused a large shift in the PC1 dimension to more positive values and significantly reduced the differences between NL and HL samples in PC2.

To examine whether chloroplasts are affected by the loss of Executer, the effect of light was investigated on the soluble chloroplast proteome of *Arabidopsis*. First, plants were grown at 120μmol m^-2^ s^-1^. After 8weeks, plants were exposed to high light intensity (700μmol m^-2^ s^-1^) during 6h and chloroplasts were isolated from rosette leaves. The soluble protein extract from chloroplasts was analysed by 2D-DIGE, following a strategy as depicted in Table 1. The DIGE analysis revealed significant differences between WT and mutant plants, both under normal light (WTNL, *ex1*NL and *ex2*NL, respectively) and in response to high light (WTHL, *ex1*HL and *ex2*HL, respectively). The differences in the stroma proteome between WT, *ex1* and *ex2* are supported by PCA (Fig. 1). Based on the first principal component (PC1)—that represents the direction of highest variability after gel data dimensionality reduction—there is a large variation between WT in normal conditions versus high light treatment. Differences between *executer* mutants and WT under normal conditions are also evident (Fig. 1).

### Proteome remodelling as a consequence of executer loss of function resembles light treatment in WT

Comparative analysis of WT and *ex1* and *ex2 Arabidopsis* plants in the two light conditions (NL and HL) showed a number of soluble chloroplast proteins that significantly altered their expression level (Supplementary Table S1; fold-change >2, p<0.001). The majority of the observed changes in the proteome were upregulations, irrespectively of genotype or treatments, with the largest changes detected in *ex2* plants.

**Fig. 2.**
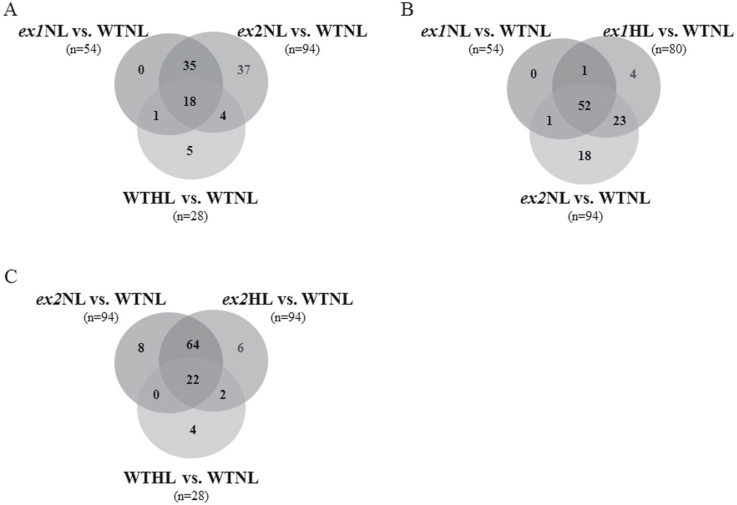
Venn diagram analyses showing common and differential distribution of protein spots detected by DIGE in stromal preparations of *Arabidopsis* plants in response to light and genotype, using as reference WT in normal growth light conditions (WTNL). (A) Overlay of the responsive proteins in WT upon light treatment (WTHL), and the two *executer* mutants in normal growth light conditions (*ex1*NL and *ex2*NL, respectively); (B) Overlay of the responsive proteins in *ex1* plants upon light treatment (*ex2*HL) and the two executer mutants in normal growth light conditions (*ex1*NL and *ex2*NL, respectively); (C) Overlay of the responsive proteins in WT upon light treatment (WTHL) and the *ex2* mutants both in normal light (*ex2*NL) and high light (*ex2*HL) conditions.

In NL conditions, changes in 54 and 94 protein spots were detected in the *ex1*NL versus WTNL and *ex2*NL versus WTNL comparative groups, respectively (Supplementary Table S1). When the responsive spots of the two mutant plants in NL versus WTNL were compared, 41 spots (37+4) were exclusively detected in *ex2*NL plants, as shown in a Venn diagram analysis in Fig. 2A. Upon exposure of WT *Arabidopsis* plants to high light (WTHL), 28 protein spots (5+1+18+4) displayed significant expression changes in response to the treatment (Supplementary Table S1). Actually, 23 light-responsive spots, which represent more than 82%, were detected in the *ex1*NL vs. WTNL and/or *ex2*NL vs. WTNL groups (1+18+4 in Fig. 2A). When *ex1* plants were exposed to high light for 6h (*ex1*HL), 27 protein spots (23+4 in Fig. 2B) were found to be differentially expressed relative to *ex1*NL. Interestingly, the behaviour of 23 spots (not present in *ex1*NL) was shared between *ex1*HL and *ex2*NL groups (Fig. 2B). Few differences were detected between *ex2*NL versus WTNL and *ex2*HL versus WTNL groups, with more than 91% common protein spots (Fig. 2C). In general, it was observed that a higher number of unique spots were significantly altered in *ex2* plants compared to WT, and the changes were already significant in normal light conditions (37 and 18 spots in Figs 2A and 2B, respectively).

### Identification and analysis of differentially expressed proteins

**Fig. 3.**
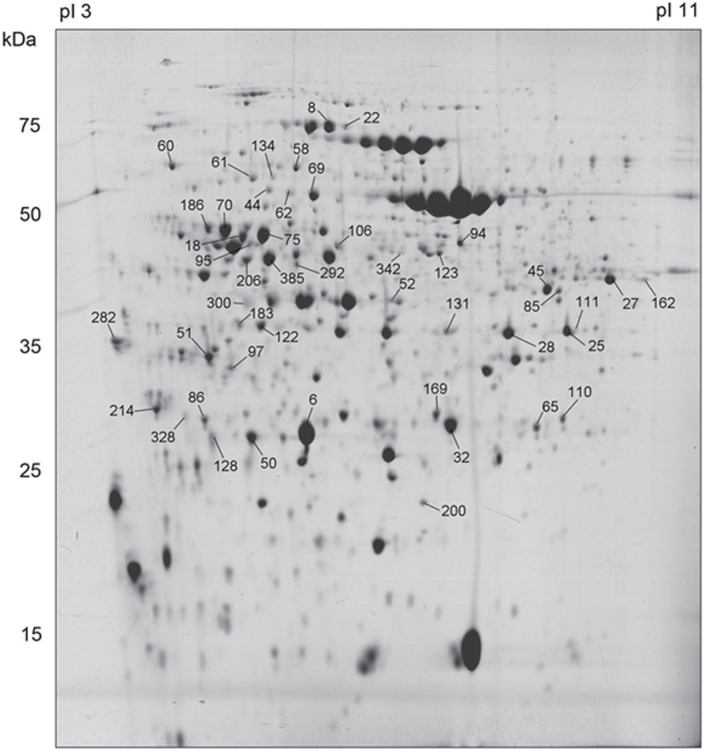
Preparative CBB-stained 2D gel of the soluble chloroplast proteome of *Arabidopsis*. The positions and numbers of the 47 identified protein spots are indicated according to the numbering in Table 3.

**Fig. 4.**
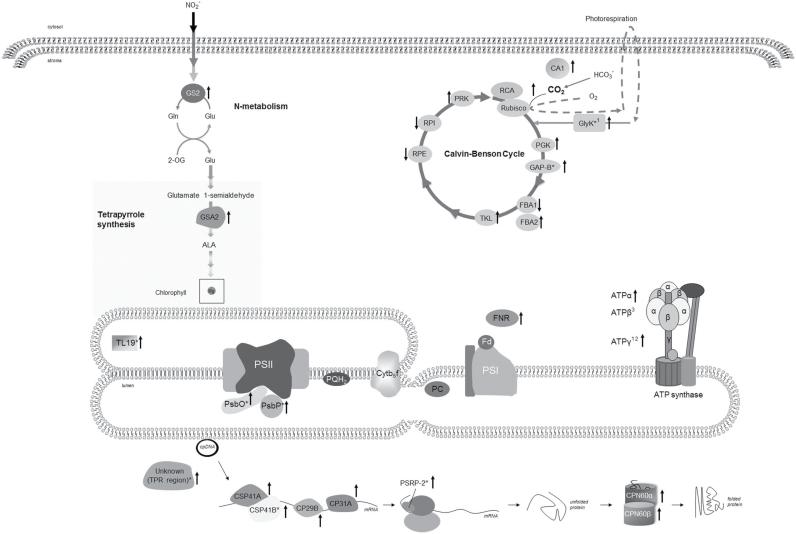
Schematic presentation of chloroplast proteins affected in *executer* mutants and WT plants after light treatment compared to WT plants under normal or growth light conditions, as shown in Table 3. Arrows indicate increased or decreased abundance compared to WTNL. An asterisk indicates that the protein is not differentially expressed in WTHL; ^1^protein is not differentially expressed in *ex1*NL, ^2^protein is not differentially expressed in *ex2*NL; ^3^multi-expression pattern. Abbreviations: ALA, 5-aminolevulinic acid; ATPα, ATPβ and ATPγ, ATPase alpha, beta and gamma subunits, respectively; CA1, carbonic anhydrase 1; CP29B’, chloroplast ribonucleoprotein 29kDa; CP31A, 31 kDA RNA-binding protein; CPN60α and CPN60β, chaperonin 60 alpha and beta subunits, respectively; CSP41A and CSP41B, chloroplast stem-loop binding protein of 41kDa A and B subunits, respectively; Cytb_6_f, cytochrome b_6_f complex; FBA1 and FBA2, ructose-bisphosphate aldolase subunits 1 and 2, respectively; Fd, ferredoxin; FNR2, ferredoxin-NADP(+)-oxidoreductase 2; GAPB, glyceraldehyde 3-phosphate dehydrogenase B subunit; Glu, glutamate; Gln, glutamine; GlyK, D-glycerate 3-kinase; GS2, glutamine synthetase-2; GSA2, glutamate-1-semialdehyde 2 1-aminomutase); PC, plastocyanin; PGK, phosphoglycerate kinase; PQH_2_, plastoquinol; PRK, phosphoribulokinase; PsbO and PsbP, photosystem II subunits O and P, respectively; PSI, photosystem I; PSII, photosystem II; PSRP-2, plastid-specific ribosomal protein 2; RCA, rubisco activase; RPE, ribulose-phosphate-3-epimerase; RPI, ribose 5-phosphate isomerase; TKL, transketolase; TL19, thylakoid lumen 19kDa; 2-OG, 2-oxoglutarate.

Based on quantitative image analysis, 107 spots were detected to be significantly altered in their accumulation among genotypes and treatments and were selected as highly responsive proteins. Ninety differentially expressed protein spots were successfully excised from colloidal Coomassie stained gels (a representative gel is shown in Fig. 3) and analysed by mass spectrometry. Gel spots corresponding to protein mixtures or albumin were discarded. Several proteins were presented as multiple spots that likely represent different isoforms or proteins differentially modified in the protein extracts or degradation products. Finally, a total of 47 spots corresponding to 29 proteins were unambiguously identified (Table 3, Fig. 3 and Supplementary Table S2). The identified proteins fell into several functional groups: photosynthesis and carbon fixation, energy generation, RNA metabolism, protein folding, and other metabolic processes, such as nitrogen and tetrapyrrole metabolism (Fig. 4). There were also some proteins of unknown function.

**Table 3.**
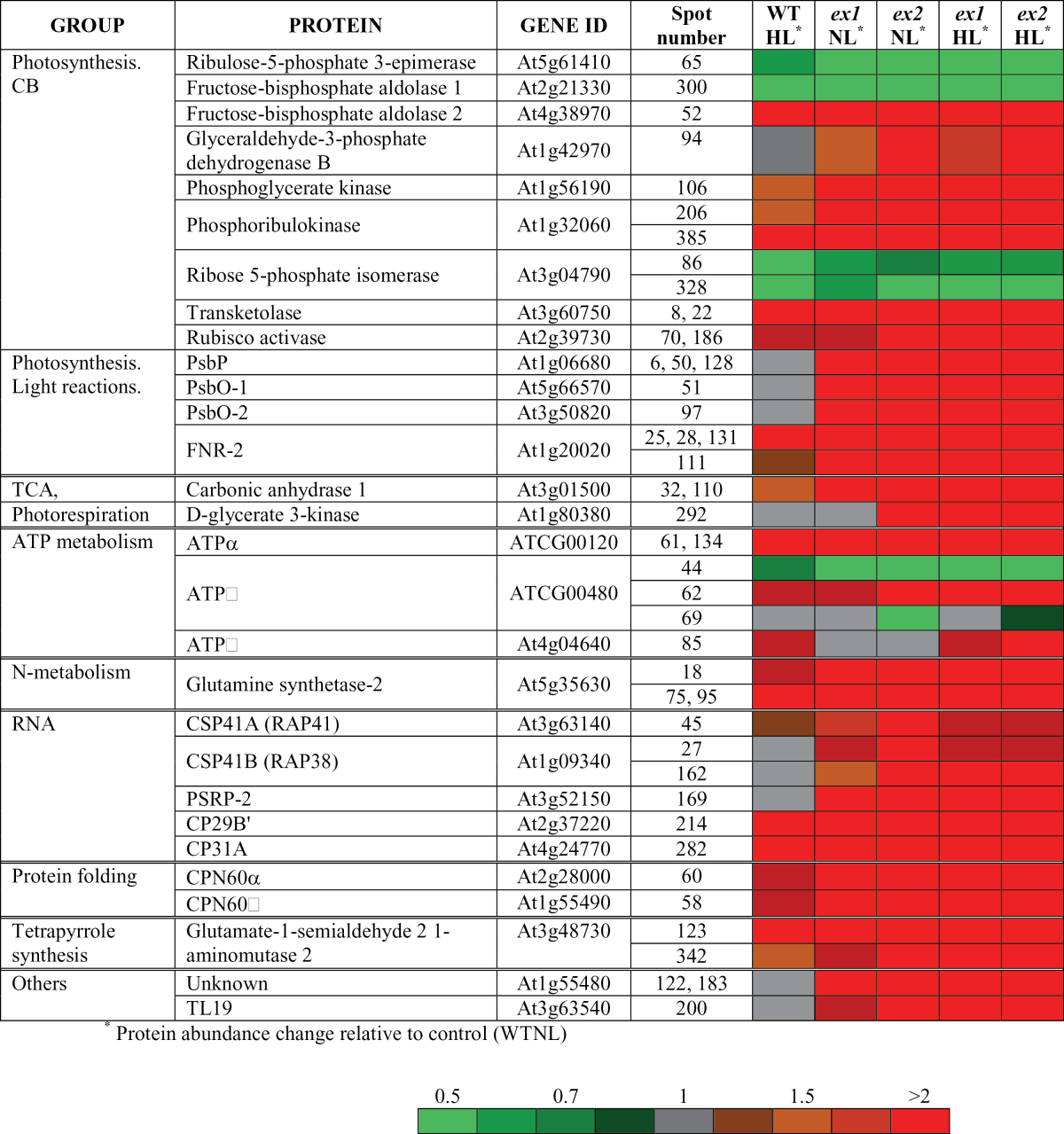
Summary of differentially expressed proteins (light treatment and/or genotype effect versus WTNL) identified by MS. The functional classification (GROUP) and gene accession number (GENE ID) are shown. The fold change in protein abundance is indicated in colour code; values >1 or <1 indicate an increase or decrease in protein abundance, respectively. A colour version of this figure is available at JXB online

### Photosynthetic electron transport chain

Ferredoxin-NADP reductase isoform 2 (FNR-2), which mediates the transferring of electrons from ferredoxin to NADP, and two proteins of the oxygen evolving complex (OEC) from photosystem II—PsbO isoforms (PsbO1 and PsbO-2) and PsbP—were more abundant in the chloroplast soluble fraction of *executer* mutant plants compared to wild type *Arabidopsis*. Except for OEC proteins, a similar trend was observed in WTHL. Two peripheral ATP synthase subunits (alpha and beta) were significantly affected in *executer* mutants: an increase abundance of the alpha subunit (ATP-A) in the stromal compartment was detected, but a multi-expression pattern (spots 44, 62 and 69) was observed for the beta subunit (ATP-B) and therefore it could not be classified simply into the up- or down-regulated group. On the other hand, the stromal ATP synthase delta subunit (ATP-C) accumulated in a light-dependent manner.

### Primary and secondary metabolism

Six Calvin-Benson (CB) enzymes (transketolase, glyceraldehyde-3-phosphate dehydrogenase B, phosphoglycerate kinase, phosphoribulokinase, rubisco activase and fructose-biphosphate aldolase 2) accumulated in mutant plants compared with WTNL. However, ribulose-5-phosphate 3-epimerase, ribose 5-phosphate isomerase and fructose-biphosphate aldolase 1 were down-regulated. For these enzymes, except glyceraldehyde-3-phosphate dehydrogenase B, a similar direction of response was detected in WTHL. The stromal enzymes glutamine synthetase-2 (involved in nitrogen metabolism) and glutamate-1-semialdehyde 2,1-aminomutase (tetrapyrrole synthesis) were upregulated in a genotype- and treatment-manner. Carbonic anhydrase 1 showed increased relative abundance in mutant plants, and also a slight increase upon light treatment. Glycerate 3-kinase, a participant of the photorespiratory cycle, was particularly affected in *ex2* mutant plants.

### Chloroplast protein synthesis and homeostasis

Alpha and beta subunits of Cpn60—a molecular chaperone that participates in protein folding in chloroplasts—increased in *executer* mutants plants and, to a lesser extent, in WTHL as compared with WTNL. Interestingly, a group of proteins related to mRNA metabolism—the chloroplast stem-loop binding proteins of 41kDa (CSP41A, CSP41B), and plastid-specific ribosomal protein 2 (PSRP-2)—were significantly upregulated in mutant plants. The accumulation of two chloroplast ribonucleoproteins, namely CP29B’ and CP31A, was affected both by genotype and light treatment.

### Other

The abundance of two chloroplast proteins of unknown function (Table 3) varied in a genotype-dependent manner. Spots 122 and 183 corresponded to a protein containing a tetratricopeptide repeat (TPR) region based on protein sequence analysis (Ishikawa *et al.*, 2005). Spot 200 was identified as a thylakoid lumen protein of 19kDa (TL19) (Schubert *et al.*, 2002).

## Discussion

Plants are exposed to a variety of environmental changes, such as light availability, that can compromise their metabolism, growth and development. Therefore, plants have developed a variety of mechanisms for sensing environmental fluctuations and, accordingly, adjust their developmental programs, metabolic processes and defence reactions. Upon changing light conditions, plants exhibit adaptation and acclimation strategies in order to optimize their photosynthetic performance and to avoid imbalance between energy absorption and utilization, which could promote ROS production in the chloroplasts (Apel, 2004; Kangasjärvi *et al.*, 2009; Karpinski *et al.*, 2013). The comparison of a five-fold increase in PFD over growth intensity, not enough for light-saturated photosynthesis, was chosen to challenge the acclimation response of WT and *executer* mutant *Arabidopsis* plants to irradiance. Chlorophyll fluorescence measurements performed under different conditions showed that the maximum quantum efficiency of PSII photochemistry remained unchanged between WT and mutants, with a moderate decrease in plants under moderate light intensity. Similar effects were detected in the light-dependent thermal dissipation component of NPQ and photochemical efficiency. It was concluded that the photochemical activity of *executer* plants was not affected relative to WT plants. Moreover, there was no significant loss of PSII efficiency during the chosen exposure time to increased light. Therefore, our experimental conditions would not promote photo-oxidative stress, but likely rapid adjustments in photosynthesis and chloroplast composition for acclimation to a change in the light environment. A double purpose is envisaged, that is, oxidative stress avoidance and molecular adjustments that would facilitate plants utilizing the additional light to improve their photosynthetic performance.

The acclimation response would involve changes in the relative abundance of a number of proteins (Kosová *et al.*, 2011). Therefore, a subcellular fractionation approach in combination with 2D-DIGE was followed to analyse the dynamics of the soluble chloroplast proteome of *Arabidopsis* to changing light. It has been shown that Executer proteins are necessary to transmit the signal produced by singlet oxygen from the plastid to the nucleus. Yet little is known about the role that Executers play under normal circumstances. In order to investigate the putative contribution of the Executer pathway to the plant response, a comparative proteome analysis of soluble chloroplast extracts of the two *executer* mutants was carried out. Our results showed very similar molecular phenotypes between *ex1* and *ex2* under growth light conditions, though the change was more significant in the latter. Interestingly, a clear separation among the WT and *executer* mutant groups was observed when plants were challenged with increased light. As expected, light treatment significantly affected the soluble chloroplast proteome in WT, but the effect was less pronounced in mutant plants, that otherwise show more similarities to WTHL. The consistent phenotype observed in our proteomic experiments for the two *executer* mutant plants indicates that Ex1 and Ex2 might participate in the same signalling pathway. Moreover, as previously proposed from genetic studies, their functions might not be redundant as the presence of one protein cannot compensate the absence of the other.

Examination of differentially accumulated proteins revealed that the proteins that underwent differential expression upon light treatment in WT *Arabidopsis* were related to metabolic pathways (mainly carbon metabolism), protein synthesis and energy production (Fig. 4). We detected an increase in abundance of FNR-2, which has a critical role in the redistribution of photosynthetically derived electrons to various reducing pathways, such as carbon fixation, nitrogen metabolism and chlorophyll biosynthesis (Lintala *et al.*, 2009). Also, the abundance of three peripheral thylakoid ATP synthase subunits was responsive to light; it has been shown that ATP synthase in chloroplasts is regulated by light and metabolite factors, and particularly ROS showed a direct influence in its activity (Buchert and Forreiter, 2010; Buchert *et al.*, 2012; Kohzuma *et al.*, 2013). Our results showed the alteration of a number of CB enzymes that catalyse readily reversible reactions and are not susceptible to ‘fine’ regulation, such as aldolase, transketolase, epimerase and isomerase (Raines, 2003; Michelet *et al.*, 2013). In our experiments, a different expression pattern for two aldolase isoforms (FBA1 and FBA2) was detected, which could indicate some functional specialization. The importance of aldolase and transketolase activities in photosynthetic carbon flux control and for the acclimation of photosynthesis to changing environmental conditions has been reported (Haake *et al.*, 1998, 1999; Henkes *et al.*, 2001; Raines, 2003; Uematsu *et al.*, 2012). Furthermore, their substrates and products act as precursors of associated metabolic processes, such as amino acid and fatty acid synthesis, and therefore variations in the abundance would affect other chloroplast pathways (Henkes *et al.*, 2001; Tetlow *et al.*, 2005). On the other hand, epimerase and isomerase decreased significantly in response to light. Our study showed that phosphoribulokinase (PRK), which plays an important role in regulating the flow of sugar through the Calvin cycle, was upregulated by light. PRK can become limiting when plants grown under low irradiance are exposed to high light (Paul *et al.*, 2000). Two enzymes with a defined role in carbon fixation modulation, rubisco activase (Portis, 2003) and beta-carbonic anhydrase 1 (CA1) (Fett and Coleman, 1994), also increased upon light treatment. Other metabolic proteins such as glutamine synthetase-2—a central enzyme in nitrogen metabolism with a role in maintaining the balance of carbon and nitrogen (Miflin and Habash, 2002)—and a GSA aminotransferase—that participates in tetrapyrrole metabolism (Tanaka *et al.*, 2011)—were induced. Increased level of proteins related to protein folding and RNA is in accordance with increased rate of protein synthesis in the light, when the translational machinery of the chloroplast is most active (Marín-Navarro *et al.*, 2007). All these changes are likely associated with an increase in the efficiency of photosynthesis under non-saturating light conditions that modify the photosynthetic capacity of the plant with an impact in carbon fixation, chlorophyll synthesis, nitrogen metabolism and other processes, and would result in new ATP requirements.

Expression of most of the proteins discussed above also changed in *executer* mutants under normal growth conditions. Further, some spots exhibited abundance variations only linked to the *Arabidopsis* genotype and were not regulated by light. Two CB enzymes (GapB and phosphoglycerate kinase) significantly increased in the mutant plants, particularly in *ex2* plants, in parallel to the alterations of CB enzymes detected upon light treatment. A subset of proteins of OEC accumulated in the soluble fraction of *executer* chloroplasts compared to WT (Ettinger and Theg, 1991; Bricker *et al.*, 2012). Two ribonucleases from the CSP41 family (Qi *et al.*, 2012) and a plastid-specific ribosomal protein (PSRP-2) (Yamaguchi and Subramanian, 2003) were more abundant in *executer* plants, which might indicate a modified transcriptional and/or translational activity compared to WT under normal growth light conditions. Two chloroplast proteins of unknown function—a protein with a predicted TPR motif and a thylakoid lumen protein that belongs to PsbP-superfamily—showed higher expression levels in mutant plants.

Our results revealed significant differences between WT and mutant plants, but an interesting overlap was found between WTHL and *executer* plants. Although Executer proteins are dispensable for normal growth, our work has detected a molecular perturbation at the basal level in *executer* mutant plants. It seems that the loss of function of Executer results in a reorientation in chloroplast central metabolism that resembles the activation response of moderate light acclimation. A plausible interpretation of these data is that the Executer proteins set the light intensity threshold that triggers the high light response. Therefore, in the *executer* mutants, this threshold is lower than in wild type plants. It is thus proposed that Executer form part of a regulatory network for the coordination between environmental stimuli and metabolic adapattion and determine the acclimation response in chloroplasts, although the exact role of the Executer proteins is yet to be defined. Interestingly, our results showed that the absence of Ex2 has a stronger impact in chloroplasts than Ex1. Our findings are consistent with other studies that observed an Executer-dependent stress acclimation in green leaves of mature plants, such as suppression of cell death in *ex1* plants treated with DCMU (Wagner *et al.*, 2004), the resistance to photoxidative stress of *ex1* plants (Ramel *et al.*, 2012), or the slight increase to pathogen resistance in *ex2* plants (Mur *et al.*, 2010).

In conclusion, despite the demonstration that Executer proteins are necessary to transmit the signal produced by singlet oxygen from the plastid to the nucleus, little is known about the role that Executers play under normal circumstances. In an effort to elucidate the biological activity of Executer proteins as putative mediators of the singlet oxygen response in chloroplasts, we used proteomics to analyse the role of Executer in chloroplasts of *Arabidopsis* exposed to different light regimes. The analysis of the soluble chloroplast protein profile in WT has provided a deeper insight into changes associated with the acclimation response to light. Six hours of high light exposure triggered responses in chloroplast of WT *Arabidopsis*; not surprisingly, many proteins involved in photosynthesis and carbon metabolism were affected, which reflects an increase in the efficiency of photosynthesis. Under normal light growth conditions, our experiments detected significant changes in the soluble chloroplast proteome as a consequence of the loss of function of Executers that, interestingly, resembled the acclimation response of the plant to increased light. Our results suggest that Executer proteins form part of the signalling network for the perception of environmental perturbation in plants, and might participate in the basal repression of defence responses in chloroplasts under normal irradiance.

## Supplementary material

Supplementary material is available at *JXB* online.

Supplementary Fig. S1. Verification of two *A. thaliana* T-DNA lines homozygous for an insertion within *EX1* and *EX2*, respectively.

Supplementary Table S1. Number of spots differentially expressed (up or down-regulated) in response to genotype (WT, ex1 and ex2 plants) and light treatment (NL and HL).

Supplementary Table S2. List of differentially expressed proteins identified by mass spectrometry in the SwissProt/TAIR9 databases.

## Abbreviations:

CB: Calvin-Benson cycle
CBB: Coomasie Brilliant Blue
DIGE: differential gel electrophoresis
Ex: Executer
HL: high light
NL: normal light
NPQ: non-photochemical quenching
PCA: principal component analysis
PFD: photon flux density
ROS: reactive oxygen species
2D: two-dimensional
WT: wild-type.

## References

[CIT0001] Alonso JM Stepanova AN Leisse TJ 2003 Genome-wide insertional mutagenesis of *Arabidopsis thaliana* . Science 301, 653–657. 12893945 10.1126/science.1086391

[CIT0002] Apel K 2004 Reactive oxygen species: metabolism, oxidative stress, and signal transduction. Annual Review of Plant Biology 55, 373–399. 10.1146/annurev.arplant.55.031903.141701 15377225

[CIT0003] Asada K 2006 Production and scavenging of reactive oxygen species in chloroplasts and their functions. Plant Physiology 141, 391–396. 16760493 10.1104/pp.106.082040 PMC1475469

[CIT0004] Barajas-López JdD Blanco NE Strand Å 2013 Plastid-to-nucleus communication, signals controlling the running of the plant cell. Biochimica et Biophysica Acta (BBA) – Molecular Cell Research 1833, 425–437. 22749883 10.1016/j.bbamcr.2012.06.020

[CIT0005] Baruah A Šimková K Apel K Laloi C 2009 *Arabidopsis* mutants reveal multiple singlet oxygen signaling pathways involved in stress response and development. Plant Molecular Biology 70, 547–563. 19449151 10.1007/s11103-009-9491-0

[CIT0006] Bilger W Björkman O 1990 Role of the xanthophyll cycle in photoprotection elucidated by measurements of light-induced absorbance changes, fluorescence and photosynthesis in leaves of *Hedera canariensis* . Photosynthesis Research 25, 173–185. 24420348 10.1007/BF00033159

[CIT0007] Bradford MM 1976 A rapid and sensitive method for the quantitation of microgram quantities of protein utilizing the principle of protein-dye binding. Analytical Biochemistry 72, 248–254. 942051 10.1016/0003-2697(76)90527-3

[CIT0008] Bricker TM Roose JL Fagerlund RD Frankel LK Eaton-Rye JJ 2012 The extrinsic proteins of Photosystem II. Biochimica et Biophysica Acta (BBA) – Bioenergetics 1817, 121–142. 21801710 10.1016/j.bbabio.2011.07.006

[CIT0009] Buchert F Forreiter C 2010 Singlet oxygen inhibits ATPase and proton translocation activity of the thylakoid ATP synthase CF1CFo. FEBS Letters 584, 147–152. 19925794 10.1016/j.febslet.2009.11.040

[CIT0010] Buchert F Schober Y Römpp A Richter ML Forreiter C 2012 Reactive oxygen species affect ATP hydrolysis by targeting a highly conserved amino acid cluster in the thylakoid ATP synthase γ subunit. Biochimica et Biophysica Acta (BBA) – Bioenergetics 1817, 2038–2048. 22727877 10.1016/j.bbabio.2012.06.007

[CIT0011] Chi W Sun X Zhang L 2013 Intracellular signaling from plastid to nucleus. Annual Review of Plant Biology 64, 559–582. 10.1146/annurev-arplant-050312-120147 23394498

[CIT0012] Estavillo GM Crisp PA Pornsiriwong W Wirtz M Collinge D Carrie C Giraud E Whelan J David P Javot H Brearley C Hell R Marin E Pogson BJ 2011 Evidence for a SAL1-PAP chloroplast retrograde pathway that functions in drought and high light signaling in *Arabidopsis* . The Plant Cell Online 23, 3992–4012. 10.1105/tpc.111.091033 PMC3246320 22128124

[CIT0013] Ettinger WF Theg SM 1991 Physiologically active chloroplasts contain pools of unassembled extrinsic proteins of the photosynthetic oxygen-evolving enzyme complex in the thylakoid lumen. The Journal of Cell Biology 115, 321–328. 1918144 10.1083/jcb.115.2.321 PMC2289146

[CIT0014] Fett JP Coleman JR 1994 Characterization and expression of two cDNAs encoding carbonic anhydrase in *Arabidopsis thaliana* . Plant Physiology 105, 707–713. 7520589 10.1104/pp.105.2.707 PMC159412

[CIT0015] Foyer CH Noctor G 2009 Redox regulation in photosynthetic organisms: signaling, acclimation, and practical implications. Antioxidants & Redox Signaling 11, 861–905. 19239350 10.1089/ars.2008.2177

[CIT0016] Fryer MJ Oxborough K Mullineaux PM Baker NR 2002 Imaging of photo‐oxidative stress responses in leaves. Journal of Experimental Botany 53, 1249–1254. 11997373

[CIT0017] Gadjev I Vanderauwera S Gechev TS Laloi C Minkov IN Shulaev V Apel K Inzé D Mittler R Van Breusegem F 2006 Transcriptomic footprints disclose specificity of reactive oxygen species signaling in Arabidopsis. Plant Physiology 141, 436–445. 16603662 10.1104/pp.106.078717 PMC1475436

[CIT0018] Galvez-Valdivieso G Fryer MJ Lawson T Slattery K Truman W Smirnoff N Asami T Davies WJ Jones AM Baker NR Mullineaux PM 2009 The high light response in Arabidopsis involves ABA signaling between vascular and bundle sheath cells. The Plant Cell Online 21, 2143–2162. 10.1105/tpc.108.061507 PMC2729609 19638476

[CIT0019] Galvez-Valdivieso G Mullineaux PM 2010 The role of reactive oxygen species in signalling from chloroplasts to the nucleus. Physiologia Plantarum 138, 430–439. 20028481 10.1111/j.1399-3054.2009.01331.x

[CIT0020] Genty B Briantais J-M Baker NR 1989 The relationship between the quantum yield of photosynthetic electron transport and quenching of chlorophyll fluorescence. Biochimica et Biophysica Acta (BBA) – General Subjects 990, 87–92.

[CIT0021] Gollnick K 2007 Type II photooxygenation reactions in solution. Advances in Photochemistry . John Wiley & Sons, Inc., pp 1–122.

[CIT0022] Haake V Geiger M Walch-Liu P Of Engels C Zrenner R Stitt M 1999 Changes in aldolase activity in wild-type potato plants are important for acclimation to growth irradiance and carbon dioxide concentration, because plastid aldolase exerts control over the ambient rate of photosynthesis across a range of growth conditions. The Plant Journal 17, 479–489.

[CIT0023] Haake V Haake V Zrenner R Sonnewald U Stitt M 1998 A moderate decrease of plastid aldolase activity inhibits photosynthesis, alters the levels of sugars and starch, and inhibits growth of potato plants. The Plant Journal 14, 147–157. 9628012 10.1046/j.1365-313x.1998.00089.x

[CIT0024] Hall M Mishra Y Schröder W 2011 Preparation of stroma, thylakoid membrane, and lumen fractions from *Arabidopsis thaliana* chloroplasts for proteomic analysis. In: Jarvis RP, ed. Chloroplast Research in Arabidopsis, 775. Humana Press, pp 207–222. 10.1007/978-1-61779-237-3_11 21863445

[CIT0025] Henkes S Sonnewald U Badur R Flachmann R Stitt M 2001 A small cecrease of plastid transketolase activity in antisense tobacco transformants has dramatic effects on photosynthesis and phenylpropanoid metabolism. The Plant Cell Online 13, 535–551. 10.1105/tpc.13.3.535 PMC135503 11251095

[CIT0026] Ishikawa A Tanaka H Kato C Iwasaki Y Asahi T 2005 Molecular characterization of the *ZKT* gene encoding a protein with PDZ, K-Box, and TPR motifs in *Arabidopsis* . Bioscience, Biotechnology, and Biochemistry 69, 972–978. 10.1271/bbb.69.972 15914918

[CIT0027] Kangasjärvi S Nurmi M Tikkanen M Aro E-M 2009 Cell-specific mechanisms and systemic signalling as emerging themes in light acclimation of C3 plants. Plant, Cell and Environment 32, 1230–1240. 10.1111/j.1365-3040.2009.01982.x 19344335

[CIT0028] Karpinski S Reynolds H Karpinska B Wingsle G Creissen G Mullineaux P 1999 Systemic signaling and acclimation in response to excess excitation energy in *Arabidopsis* . Science 284, 654–657. 10213690 10.1126/science.284.5414.654

[CIT0029] Karpinski S Szechynska-Hebda M Wituszynska W Burdiak P 2013 Light acclimation, retrograde signalling, cell death and immune defences in plants. Plant Cell and Environment 36, 736–744. 10.1111/pce.12018 23046215

[CIT0030] Kim C Apel K 2013 *a* ^1^O_2_-mediated and EXECUTER-dependent retrograde plastid-to-nucleus signaling in norflurazon-treated seedlings of *Arabidopsis thaliana* . Molecular Plant 6, 1580–1591. 23376773 10.1093/mp/sst020 PMC3842135

[CIT0031] Kim C Apel K 2013 *b* Singlet oxygen-mediated signaling in plants: moving from flu to wild type reveals an increasing complexity. Photosynthesis Research 116, 455–464. 23832611 10.1007/s11120-013-9876-4 PMC3833438

[CIT0032] Kim C Lee KP Baruah A Nater M Göbel C Feussner I Apel K 2009 1O2-mediated retrograde signaling during late embryogenesis predetermines plastid differentiation in seedlings by recruiting abscisic acid. Proceedings of the National Academy of Sciences 106, 9920–9924. 10.1073/pnas.0901315106 PMC2701023 19482940

[CIT0033] Kim C Meskauskiene R Zhang S Lee KP Lakshmanan Ashok M Blajecka K Herrfurth C Feussner I Apel K 2012 Chloroplasts of *Arabidopsis* are the source and a primary target of a plant-specific programmed cell death signaling pathway. The Plant Cell Online 24, 3026–3039. 10.1105/tpc.112.100479 PMC3426130 22797473

[CIT0034] Kohzuma K Dal Bosco C Meurer J Kramer DM 2013 Light- and metabolism-related regulation of the chloroplast ATP synthase has distinct mechanisms and functions. Journal of Biological Chemistry 288, 13156–13163. 23486473 10.1074/jbc.M113.453225 PMC3642356

[CIT0035] Kosová K Vítámvás P Prášil IT Renaut J 2011 Plant proteome changes under abiotic stress—Contribution of proteomics studies to understanding plant stress response. Journal of Proteomics 74, 1301–1322. 21329772 10.1016/j.jprot.2011.02.006

[CIT0036] Koussevitzky S Nott A Mockler TC Hong F Sachetto-Martins G Surpin M Lim J Mittler R Chory J 2007 Signals from chloroplasts converge to regulate nuclear gene expression. Science 316, 715–719. 17395793

[CIT0037] Krieger-Liszkay A Fufezan C Trebst A 2008 Singlet oxygen production in photosystem II and related protection mechanism. Photosynthesis Research 98, 551–564. 18780159 10.1007/s11120-008-9349-3

[CIT0038] Laloi C Przybyla D Apel K 2006 A genetic approach towards elucidating the biological activity of different reactive oxygen species in *Arabidopsis thaliana* . Journal of Experimental Botany 57, 1719–1724. 16720605 10.1093/jxb/erj183

[CIT0039] Laloi C Stachowiak M Pers-Kamczyc E Warzych E Murgia I Apel K 2007 Cross-talk between singlet oxygen- and hydrogen peroxide-dependent signaling of stress responses in *Arabidopsis thaliana* . Proceedings of the National Academy of Sciences 104, 672–677. 10.1073/pnas.0609063103 PMC1766442 17197417

[CIT0040] Ledford HK Chin BL Niyogi KK 2007 Acclimation to singlet oxygen stress in *Chlamydomonas reinhardtii* . Eukaryotic Cell 6, 919–930. 17435007 10.1128/EC.00207-06 PMC1951523

[CIT0041] Lee KP Kim C Landgraf F Apel K 2007 EXECUTER1- and EXECUTER2-dependent transfer of stress-related signals from the plastid to the nucleus of *Arabidopsis thaliana* . Proceedings of the National Academy of Sciences 104, 10270–10275. 10.1073/pnas.0702061104 PMC1891253 17540731

[CIT0042] Lintala M Allahverdiyeva Y Kangasjärvi S Lehtimäki N Keränen M Rintamäki E Aro EM Mulo P 2009 Comparative analysis of leaf-type ferredoxin-NADP+ oxidoreductase isoforms in *Arabidopsis thaliana* . Plant Journal 57, 1103–1115. 19054362 10.1111/j.1365-313X.2008.03753.x

[CIT0043] Marín-Navarro J Manuell A Wu J, P. Mayfield S 2007 Chloroplast translation regulation. Photosynthesis Research 94, 359–374. 17661159 10.1007/s11120-007-9183-z

[CIT0044] Meskauskiene R Nater M Goslings D Kessler F op den Camp R Apel K 2001 FLU: A negative regulator of chlorophyll biosynthesis in *Arabidopsis thaliana* . Proceedings of the National Academy of Sciences 98, 12826–12831. 10.1073/pnas.221252798 PMC60138 11606728

[CIT0045] Michelet L Zaffagnini M Morisse S Sparla F Pérez-Pérez ME Francia F Danon A Marchand C Fermani S Trost P Lemaire SD 2013 Redox regulation of the Calvin-Benson cycle: something old, something new. Frontiers in Plant Science 4, 470. 24324475 10.3389/fpls.2013.00470 PMC3838966

[CIT0046] Miflin BJ Habash DZ 2002 The role of glutamine synthetase and glutamate dehydrogenase in nitrogen assimilation and possibilities for improvement in the nitrogen utilization of crops. Journal of Experimental Botany 53, 979–987. 11912240 10.1093/jexbot/53.370.979

[CIT0047] Mur LAJ Aubry S Mondhe M Kingston Smith A Gallagher J Timms-Taravella E Timms Taravella E James C Papp I Hörtensteiner S Thomas H Ougham H 2010 Accumulation of chlorophyll catabolites photosensitizes the hypersensitive response elicited by *Pseudomonas syringae* in *Arabidopsis* . New Phytologist 188, 161–174. 20704660 10.1111/j.1469-8137.2010.03377.x

[CIT0048] Ng S De Clercq I Van Aken O Law SR Ivanova A Willems P Giraud E Van Breusegem F Whelan J 2014 Anterograde and retrograde regulation of nuclear genes encoding mitochondrial proteins during growth, development, and stress. Molecular Plant 7, 1075–1093. 24711293 10.1093/mp/ssu037

[CIT0049] Nott A Jung H-S Koussevitzky S Chory J 2006 Plastid-to-nucleus retrograde signaling. Annual Review of Plant Biology 57, 739–759. 10.1146/annurev.arplant.57.032905.105310 16669780

[CIT0050] op den Camp RGL Przybyla D Ochsenbein C Laloi C Kim C Danon A Wagner D Hideg É Göbel C Feussner I Nater M Apel K 2003 Rapid induction of distinct stress responses after the release of singlet oxygen in *Arabidopsis* . The Plant Cell Online 15, 2320–2332. 10.1105/tpc.014662 PMC197298 14508004

[CIT0051] Paul M Driscoll S Andralojc PJ 2000 Decrease of phosphoribulokinase activity by antisense RNA in transgenic tobacco: definition of the light environment under which phosphoribulokinase is not in large excess. Planta 211, 112–119. 10923711 10.1007/s004250000269

[CIT0052] Pesaresi P Hertle A Pribil M Kleine T Wagner R Strissel H Ihnatowicz A Bonardi V Scharfenberg M Schneider A Pfannschmidt T Leister D 2009 *Arabidopsis* STN7 kinase provides a link between short- and long-term photosynthetic acclimation. *The Plant Cell Online* **21,** 2402–2423. 10.1105/tpc.108.064964 PMC2751956 19706797

[CIT0053] Petrillo E Godoy Herz MA Fuchs A Reifer D Fuller J Yanovsky MJ Simpson C Brown JWS Barta A Kalyna M Kornblihtt AR 2014 A chloroplast retrograde signal regulates nuclear alternative splicing. Science 344, 427–430. 24763593 10.1126/science.1250322 PMC4382720

[CIT0054] Pfalz J Liebers M Hirth M Gruebler B Holtzegel U Schroeter Y Dietzel L Pfannschmidt T 2012 Environmental control of plant nuclear gene expression by chloroplast redox signals. Frontiers in Plant Science 3. 10.3389/fpls.2012.00257 PMC3500774 23181068

[CIT0055] Pfannschmidt T 2010 Plastidial retrograde signalling – a true ‘plastid factor’ or just metabolite signatures? Trends in Plant Science 15, 427–435. 20580596 10.1016/j.tplants.2010.05.009

[CIT0056] Pogson BJ Albrecht V 2011 Genetic dissection of chloroplast biogenesis and development: An overview. Plant Physiology 155, 1545–1551. 21330494 10.1104/pp.110.170365 PMC3091115

[CIT0057] Portis A Jr 2003 Rubisco activase – Rubisco’s catalytic chaperone. Photosynthesis Research 75, 11–27. 16245090 10.1023/A:1022458108678

[CIT0058] Przybyla D Göbel C Imboden A Hamberg M Feussner I Apel K 2008 Enzymatic, but not non-enzymatic, ^1^O_2_-mediated peroxidation of polyunsaturated fatty acids forms part of the EXECUTER1-dependent stress response program in the flu mutant of *Arabidopsis thaliana* . The Plant Journal 54, 236–248. 18182022 10.1111/j.1365-313X.2008.03409.x

[CIT0059] Qi Y Armbruster U Schmitz-Linneweber C Delannoy E de Longevialle AF Rühle T Small I Jahns P Leister D 2012 *Arabidopsis* CSP41 proteins form multimeric complexes that bind and stabilize distinct plastid transcripts. Journal of Experimental Botany 63, 1251–1270. 22090436 10.1093/jxb/err347 PMC3276088

[CIT0060] Raines C 2003 The Calvin cycle revisited. Photosynthesis Research 75, 1–10. 16245089 10.1023/A:1022421515027

[CIT0061] Ramel F Birtic S Ginies C Soubigou-Taconnat L Triantaphylidès C Havaux M 2012 Carotenoid oxidation products are stress signals that mediate gene responses to singlet oxygen in plants. Proceedings of the National Academy of Sciences 109, 5535–5540. 10.1073/pnas.1115982109 PMC3325660 22431637

[CIT0062] Rolland F Baena-Gonzalez E Sheen J 2006 Sugar sensing and signaling in plants: Conserved and novel mechanisms. Annual Review of Plant Biology 57, 675–709. 10.1146/annurev.arplant.57.032905.105441 16669778

[CIT0063] Schubert M Petersson UA Haas BJ Funk C Schröder WP Kieselbach T 2002 Proteome map of the chloroplast lumen of *Arabidopsis thaliana* . Journal of Biological Chemistry 277, 8354–8365. 11719511 10.1074/jbc.M108575200

[CIT0064] Strand A Asami T Alonso J Ecker JR Chory J 2003 Chloroplast to nucleus communication triggered by accumulation of Mg-protoporphyrinIX. Nature 421, 79–83. 12511958 10.1038/nature01204

[CIT0065] Sun X Feng P Xu X Guo H Ma J Chi W Lin R Lu C Zhang L 2011 A chloroplast envelope-bound PHD transcription factor mediates chloroplast signals to the nucleus. Nature Communications 2, 477. 10.1038/ncomms1486 21934661

[CIT0066] Tanaka R Kobayashi K Masuda T 2011 Tetrapyrrole metabolism in *Arabidopsis thaliana* . The Arabidopsis Book 9, e0145. 22303270 10.1199/tab.0145 PMC3268503

[CIT0067] Tetlow IJ Rawsthorne S Raines C Emes MJ 2005 Plastid metabolic pathways. Annual Plant Reviews 13, 60–125.

[CIT0068] Uematsu K Suzuki N Iwamae T Inui M Yukawa H 2012 Increased fructose 1,6-bisphosphate aldolase in plastids enhances growth and photosynthesis of tobacco plants. Journal of Experimental Botany 63, 3001–3009. 22323273 10.1093/jxb/ers004

[CIT0069] Wagner D Przybyla D op den Camp R Kim C Landgraf F Lee KP Würsch M Laloi C Nater M Hideg E Apel K 2004 The genetic basis of singlet oxygen induced stress responses of *Arabidopsis thaliana* . Science 306, 1183–1185. 15539603 10.1126/science.1103178

[CIT0070] Weigel D 2002 CTAB DNA miniprep. In: Curtis S, ed. Arabidopsis: a Laboratory Manual, 165 New York: Cold Spring Harbor Laboratory Press.

[CIT0071] Woodson JD Chory J 2008 Coordination of gene expression between organellar and nuclear genomes. Nature Reviews Genetics 9, 383–395. 10.1038/nrg2348 PMC4854206 18368053

[CIT0072] Woodson Jesse D Perez-Ruiz Juan M Chory J 2011 Heme synthesis by plastid ferrochelatase i regulates nuclear gene expression in plants. Current Biology 21, 897–903. 21565502 10.1016/j.cub.2011.04.004 PMC4886857

[CIT0073] Xiao Y Savchenko T Baidoo Edward EK Chehab Wassim E Hayden Daniel M Tolstikov V Corwin Jason A Kliebenstein Daniel J Keasling Jay D Dehesh K 2012 Retrograde signaling by the plastidial metabolite MEcPP regulates expression of nuclear stress-response genes. Cell 149, 1525–1535. 22726439 10.1016/j.cell.2012.04.038

[CIT0074] Yamaguchi K Subramanian AR 2003 Proteomic identification of all plastid-specific ribosomal proteins in higher plant chloroplast 30S ribosomal subunit. European Journal of Biochemistry 270, 190–205. 12605670 10.1046/j.1432-1033.2003.03359.x

[CIT0075] Zhang Z-W Yuan S Feng H Xu F Cheng J Shang J Zhang D-W Lin H-H 2011 Transient accumulation of Mg-protoporphyrin IX regulates expression of PhANGs – New evidence for the signaling role of tetrapyrroles in mature *Arabidopsis* plants. Journal of Plant Physiology 168, 714–721. 21216024 10.1016/j.jplph.2010.10.016

[CIT0076] Zhang Z-W Yuan S Xu F Yang H Zhang N-H Cheng J Lin H-H 2010 The plastid hexokinase pHXK: A node of convergence for sugar and plastid signals in *Arabidopsis* . FEBS Letters 584, 3573–3579. 20650273 10.1016/j.febslet.2010.07.024

